# Biologically consistent dose accumulation using daily patient imaging

**DOI:** 10.1186/s13014-021-01789-3

**Published:** 2021-04-06

**Authors:** Nina I. Niebuhr, Mona Splinter, Tilman Bostel, Joao Seco, Clemens M. Hentschke, Ralf O. Floca, Juliane Hörner-Rieber, Markus Alber, Peter Huber, Nils H. Nicolay, Asja Pfaffenberger

**Affiliations:** 1 Medical Physics in Radiation Oncology, German Cancer Research Center (DKFZ), Heidelberg, Germany; 2 Heidelberg Institute for Radiooncology (HIRO), National Center for Radiation Research in Oncology (NCRO), Heidelberg, Germany; 3 Department of Physics and Astronomy, Heidelberg University, Heidelberg, Germany; 4 Clinical Cooperation Unit “Radiation Oncology”, German Cancer Research Center (DKFZ), Heidelberg, Germany; 5 Department of Radiation Oncology, University Medical Center Mainz, Mainz, Germany; 6 Biomedical Physics in Radiation Oncology, German Cancer Research Center (DKFZ), Heidelberg, Germany; 7 Medical Image Computing, German Cancer Research Center (DKFZ), Heidelberg, Germany; 8 Department of Radiation Oncology, University Hospital Heidelberg, Heidelberg, Germany; 9 Molecular Radiation Oncology, German Cancer Research Center (DKFZ), Heidelberg, Germany; 10 Department of Radiation Oncology, Freiburg University Medical Center, Freiburg, Germany

**Keywords:** Dose accumulation, Linear quadratic model, Image guidance, Delivered dose, Normal tissue response, Radiobiology

## Abstract

**Background:**

This work addresses a basic inconsistency in the way dose is accumulated in radiotherapy when predicting the biological effect based on the linear quadratic model (LQM). To overcome this inconsistency, we introduce and evaluate the concept of the total biological dose, bEQD_d_.

**Methods:**

Daily computed tomography imaging of nine patients treated for prostate carcinoma with intensity-modulated radiotherapy was used to compute the delivered deformed dose on the basis of deformable image registration (DIR). We compared conventional dose accumulation (DA) with the newly introduced bEQD_d_, a new method of accumulating biological dose that considers each fraction dose and tissue radiobiology. We investigated the impact of the applied fractionation scheme (conventional/hypofractionated), uncertainties induced by the DIR and by the assigned α/β-value.

**Results:**

bEQD_d_ was systematically higher than the conventionally accumulated dose with difference hot spots of 3.3–4.9 Gy detected in six out of nine patients in regions of high dose gradient in the bladder and rectum. For hypofractionation, differences are up to 8.4 Gy. The difference amplitude was found to be in a similar range to worst-case uncertainties induced by DIR and was higher than that induced by α/β.

**Conclusion:**

Using bEQD_d_ for dose accumulation overcomes a potential systematic inaccuracy in biological effect prediction based on accumulated dose. Highest impact is found for serial-type late responding organs at risk in dose gradient regions and for hypofractionation. Although hot spot differences are in the order of several Gray, in dose-volume parameters there is little difference compared with using conventional or biological DA. However, when local dose information is used, e.g. dose surface maps, difference hot spots can potentially change outcomes of dose-response modelling and adaptive treatment strategies.

## Background

Fractionation is a key principle in the success of radiotherapy treatments, exploiting the biological advantages of different tissue radiosensitivities to open up the therapeutic window described by the commonly used linear quadratic model (LQM) for cell survival [1]. However, set-up errors and organ motion between fractions introduce discrepancies between planned and delivered dose, especially in organs at risk. Daily patient imaging facilitates the estimation of the actually delivered dose of the day. Currently, more and more studies are emerging that make use of the newly gained information for the correlation of delivered dose to toxicity [2–4].

While the fractionated treatment scheme is based on the use of the LQM and daily imaging is available to gain information about the daily dose, both principles are not yet consistently used together. Fractionated treatment according to the LQM is described by a power-to-n law which is only valid if each fraction dose is equal. In case of dose variations, it is not valid to perform a linear accumulation of the dose for the biological effect prediction due to the non-linear nature of the cell survival curve. Furthermore, important information on the daily doses is lost by averaging the fraction dose in the process of dose accumulation. In doing so, the predicted biological effect of the total treatment will potentially differ from the true biological effect according to consistent use of the dose information within the LQM.

In the absence of daily dose information there was no need to change the application of the LQM for effect prediction. The situation is changing as a result of increased use of daily imaging, and the inaccuracy in the biological effect prediction induced by dose accumulation is manifested in common practice. To overcome this inaccuracy, Zavgorodni et al. [5] introduced the concept of the equivalent constant dose, a principle to incorporate the variance of the dose into the biological effect prediction. This principle was later compared with the conventional way of effect prediction [6, 7]. Bortfeld et al. [6] evaluated the difference in cell survival prediction theoretically, finding that differences are potentially large for high dose variations, but are negligible for variations below 10% of the total dose. The difference was further studied by de Xivry et al. [7], on a set of 10 head and neck patients undergoing weekly CT scans. As the highest difference between biological and conventional dose accumulation was 2.6 Gy locally, but was far below 1 Gy for the mean organ dose, they concluded that the use of the biological model might only be relevant if local dose information is used rather than dose-volume based parameters.

Steeper dose gradients used in IMRT treatments and especially for hypofractionation might cause stronger local dose variations that become traceable by more frequent use of *daily* imaging. Furthermore, new studies exploit the daily dose information on a local scale by using dose surface maps, for example in the correlation of dose to outcome [4, 8–11]. Together, these factors emphasize the importance of correct biological model application in order to exploit the full potential of new methods and information. Despite these recent advances indicating an increasing relevance of the consistent use of biological models, the difference between conventional and biological dose accumulation has not yet been extensively studied.

In this study, we aimed to evaluate biological dose accumulation on the basis of daily CT imaging. For this, we introduce the approach of biological dose accumulation, based on the principle of the equivalent dose, for a consistent use of the biological model. We investigated the difference between common dose accumulation and the total biological dose, the latter being based on delivered deformed doses after deformable image registration on a voxel-scale. The evaluation was performed for nine prostate cancer patients who underwent daily CT scans. In the pelvic region, organ motion is a common problem between fractions, introducing high local differences between planned and delivered dose, especially in organs at risk like the bladder and rectum [12–16]. As an example, Nassef et al. [16] measured the mean dose standard deviation to the bladder to be 6.9 Gy (case maximum 18.1 Gy) and rectum to be 2.0 Gy (case maximum 4.2 Gy) in prostate IMRT. Dose differences among various dose-volume measures (V_50Gy_—V_75Gy_) varied from − 10% to + 7% for the rectum and − 4% to + 26% for the bladder in 75% of cases.

While literature mostly reports deviations of planned and delivered dose for summarizing dose volume parameters, the current analysis focusses on dose at a voxel-level from daily CT scans. The aim of this analysis was to identify scenarios where differences between the method of biological dose accumulation introduced here and the conventional method might be of interest to be taken into account in dose accumulation applications. Therefore, the impact of a range of different parameters was investigated theoretically and in patient data: the dose variation magnitude, the fractionation scheme, the α/β-value and the uncertainties from DIR.

## Methods

### Theory

#### The effect accumulation approach

According to the linear quadratic model (LQM), the cell survival fraction (SF) after a dose d for cells with radio sensitivity with the tissue specific α and β values is given by [17]:

1 $$SF=\hbox{exp}\left(-\left(\alpha d+ \beta {d}^{2}\right)\right)$$

After n fractions delivering the same dose d, the survival fraction is given by [18]:

2 $${SF}_{D}={\hbox{exp}\left(-\left(\alpha d+ \beta {d}^{2}\right)\right)}^{n}=\hbox{exp}\left(-\left(\alpha {D}_{a}+ \beta n{d}^{2}\right)\right)$$

with the total accumulated dose ${D}_{a}= \sum_{i=1}^{n}{d}_{i}=nd$. This is approximately true in regions of homogeneous dose like the target, but may not be applicable in regions of varying doses in normal tissue, especially in regions of high dose gradient. When daily information of the delivered dose is acquired, the single delivered doses d_i_ can be estimated and accumulated to the total delivered dose D_a_ that can potentially be different from the planned dose D_p_. However, the accumulation of the dose prior to the application of the LQM, as given in Eq. 2, assumes a constant mean dose over the course of therapy given by d = D_a_/n. Thus, important information about the daily variations of the doses d_i_ is lost in the process of averaging over time. In other words, by performing dose accumulation, the amount of cell kill due to radiation of previous fractions is not adequately considered, and for all following fractions the accumulation of any further fractions is based on an erroneous accumulated cell survival fraction.

To exploit the potential of daily imaging, the daily delivered doses have to be considered individually in the process of biological effect prediction. This can be achieved by deriving the SF of each treatment fraction prior to accumulation, thus, by interchanging the steps of accumulation and survival prediction:

3 $${SF}_{di}=\hbox{exp}\left(-\left(\alpha {d}_{1}+ \beta {d}_{1}^{2}\right)\right)*\hbox{ exp}\left(-\left(\alpha {d}_{2}+ \beta {d}_{2}^{2}\right)\right)*\dots *\hbox{ exp}\left(-\left(\alpha {d}_{n}+ \beta {d}_{n}^{2}\right)\right)=\hbox{exp}\left(-\left(\alpha {D}_{a}+ \beta \sum_{i=1}^{n}{d}_{i}^{2}\right)\right)$$

This approach, from now on referred to as the “effect accumulation” approach, yields the mathematically correct SF after n treatment fractions of varying doses d_i_.

The two approaches show a systematic mathematical difference in the β-term. They can be compared using Jensen’s inequality [19] for a convex function:

4 $$f\left(\sum_{i=1}^{n}\frac{1}{n}{\hbox{x}}_{i}\right)\le \sum_{i=1}^{n}\frac{1}{n}\hbox{f}({\hbox{x}}_{i})$$

with n being the number of fractions. For the convex quadratic function for daily doses d_i_, this yields:

5 $${(\sum_{i=1}^{n}\frac{1}{n}{d}_{i})}^{2}\le \sum_{i=1}^{n}\frac{1}{n}{d}_{i}^{2}$$

For the biological effect this implies that

6 $$\alpha D_a+ \beta {nd}^{2}\le \alpha D_a+ \beta \sum_{i=1}^{n}{d}_{i}^{2}$$

where ${(\sum_{i=1}^{n}\frac{1}{n}{d}_{i})}^{2}=n{d}^{2}$ with ${\sum }_{i=1}^{n}{d}_{i}=nd$.For the survival fraction it follows that:

7 $$\hbox{exp}(-\left(\alpha D_a+ \beta {nd}^{2}\right))\ge \hbox{exp}(-(\alpha D_a+ \beta \sum_{i=1}^{n}{d}_{i}^{2}))$$

Together with Eqs. 2 and 3, this gives a direct comparison between dose accumulation and effect accumulation:

8 $${SF}_{d }\ge {SF}_{di}$$

Thus, dose accumulation generally overestimates the mathematically correct cell survival fraction after n fractions in the presence of dose variations (within the context of the LQM).

#### Biological dose accumulation: the bEQD_d_-model

As derived above, the accumulation of the individual survival fractions after each treatment fraction yields a different result from the survival fraction after dose accumulation. The other way around, this suggests that the total dose of a treatment that yields the mathematically correct survival fraction according to the LQM, is different from conventionally accumulated dose.

To derive the total dose that fits the effect accumulation model, in the following called the “total biological dose”, the equieffective-dose principle is used:

The biological effect of a treatment given in constant d [Gy] per fraction is given by [20]:

9 $${E}_{d}/\alpha ={bEQD}_{d}\left(1+\frac{d}{\alpha /\beta }\right)$$

Here,${bEQD}_{d}$ represents the total treatment dose that yields the biological effect E_d_ delivered in equal doses of d. On the other hand, the effect of a treatment with varying doses d_i_ is given by:

10 $${E}_{di}/\alpha =\sum_{i}{d}_{i}+\frac{\sum_{i}{d}_{i}^{2}}{\alpha /\beta }={D}_{a}+ \frac{\sum_{i}{d}_{i}^{2}}{\alpha /\beta }$$

For conversion of a treatment with constant dose d to a biologically equivalent treatment (equieffective) with varying doses d_i_, both effects are set to be equal: ${E}_{d}/\alpha ={E}_{di}/\alpha$. With this the *total biological dose* is given by:

11 $${bEQD}_{d}({d}_{i})=\frac{\left(\alpha /\beta {D}_{a}+ \sum_{i}{d}_{i}^{2}\right)}{\alpha /\beta +d}$$

If the dose per fraction is constant, ${d}_{i}=d$, then bEQD_d_ = D_a_.

For the following, d in Eq. 5 will be set equal to the mean fraction dose, so d = D_a_/n. This way, bEQD_d_(d_i_) gives a direct comparison to the dose-accumulation-based approach by deriving the total biological dose of a treatment, with the same mean fraction dose d as D_a_.

To compare both total doses, Jensen’s inequality (Eq. 4) can again be applied and yields:

12 $${bEQD}_{d}\left({d}_{i}\right)\ge {D}_{a}$$

Thus, the total biological dose bEQD_d_(d_i_), that takes each dose per fraction into account for the biological effect, is always larger than or equal to the conventionally accumulated dose. This is in accordance with Eq. 7 that predicts that dose accumulation overestimates the cell survival fraction.

For the following analysis, this work focusses on the difference between the total dose after conventional dose accumulation ${D}_{a}$ and after biological dose accumulation, given by bEQD_d_, in order to compare the total treatment.

### Theoretical analysis

Theoretical studies were performed for a fictional voxel which receives a certain mean dose with fractional dose variations. The simulated fractionation was chosen with reference to the schemes for patient treatment: prescription dose corresponding to standard fractionation of 76.5 Gy in 2.25 Gy per fraction; hypofractionation with 63 Gy in 3.0 Gy per fraction (equivalent dose of 75.9 Gy with an α/β-value of 1.4 Gy, see next section). Four different scenarios were chosen: a voxel in a high and in a medium dose region (referring to high = prescription dose, medium = dose likely to occur in gradients in OARs), for standard and for hypofractionation, respectively, giving the following fictional voxel mean doses: standard high = 2.25 Gy × 34 fractions, standard medium: 1.5 Gy × 34 fraction, hypo high: 3.0 Gy × 21 fractions, hypo medium: 2.25 Gy × 21 fractions. The investigations focused on the dependencies of the difference between bEQD_d_ and D_a_ on the magnitude of dose variations, the assigned α/β-value, mean dose and the fractionation scheme.

All analysis was performed in R [21]. The dose variation magnitudes, given as the standard deviation are chosen to be 0%, 12.5%, 25% and 50% with respect to the mean dose, where bEQD_d_ with 0% dose variation is equal to the conventionally accumulated dose D_a_. The wide range of dose variation standard deviations was chosen to cover the observed spectrum in our patient results. In the dose gradient regions, the standard deviation varied strongly between voxels, going up to 100% in the lower dose regimes. Observed standard deviations for medium-dose voxels were around 20% in relevant numbers of voxels. Higher standard deviations, up to 50%, were chosen to investigate possible outcomes from strong organ motion and steep dose gradients.

### Patient data

Patient data were analyzed for 9 patients treated for prostate carcinoma with 6 MV IMRT (Siemens Artiste) using 9 coplanar fields. Six of the patients, undergoing definitive radiotherapy, received a standard fractionation in 34 fractions of 2.25 Gy (total physical treatment dose of 76.5 Gy). The other three patients received postoperative irradiation of the prostatic fossa with 34 fractions of 2 Gy (total treatment dose of 68 Gy). Daily CT-imaging was performed with an in-room CT scanner (CT on-rails, Siemens Somatom Emotion, located in the treatment room) prior to every treatment session, on the treatment couch and in treatment position, resulting in a total of 306 analyzed CT scans. Image guidance was performed with translational couch shifts for target position correction. Contours of the target (CTV + 7 mm setup margin) and organs at risk (OARs) were manually delineated on the treatment planning CT and on all daily CT scans by board-certified radiation oncologists (medical Council (Aerztekammer) of the German federal state Baden-Wuerttemberg, Germany). In individual cases, intersection of the delineated CTV and the bladder contour is visible. The decision for these enlarged CTV margins was individually based on a suspected locally advanced diffuse infiltration of the surrounding tissue based on the patients advanced age and tumor staging of T4. Calculation of the daily delivered doses was performed with the RayStation® treatment planning system (version 6.1.1.2), by recalculation of the original dose on the daily CT scans (in the following referred to as forward calculated dose). The study was approved by the Independent Ethics Committee of the Medical Faculty of the University of Heidelberg, Germany (S-380/2017). Further information on the study and clinical investigations based on the same data can be found in [22] and [23].

In addition to the applied treatment plans using standard fractionation in 34 fractions, hypofractionated plans were created for all patients by rescaling the physical dose. Standard EQD-conversion with α/β = 1.4 Gy for the target [24] was performed to create biologically equivalent fractionation schemes with a lower number of fractions. We used 3.0 Gy per fraction (2.7 Gy for the fossa) for moderate hypofractionation [25] given in 21 fractions leading to a total physical treatment dose of 63 Gy (equivalent to 75.9 Gy) based on EQD_2_-conversion with α/β = 1.4 Gy (it should be noted that reported α/β-values vary, the dependency of the biological dose on this will be analyzed in the uncertainty assessment below). Furthermore, strong hypofractionation was planned for 5 fractions resulting in a dose per fraction of 6.8 Gy (6.1 Gy for the fossa) with a total physical treatment dose of 34 Gy (equivalent to 76.4 Gy with a conversion based on α/β = 1.4 Gy). All dose constraints for the OARs converted using EQD were still complied with the scaled dose plans. Scaling was performed on the already deformed doses and then accumulated. The first 5 and 21 fractions, respectively, were chosen for analysis to accurately simulate the treatment as if the patient was actually treated in a shorter time.

### Data processing and analysis

**Fig. 1:**
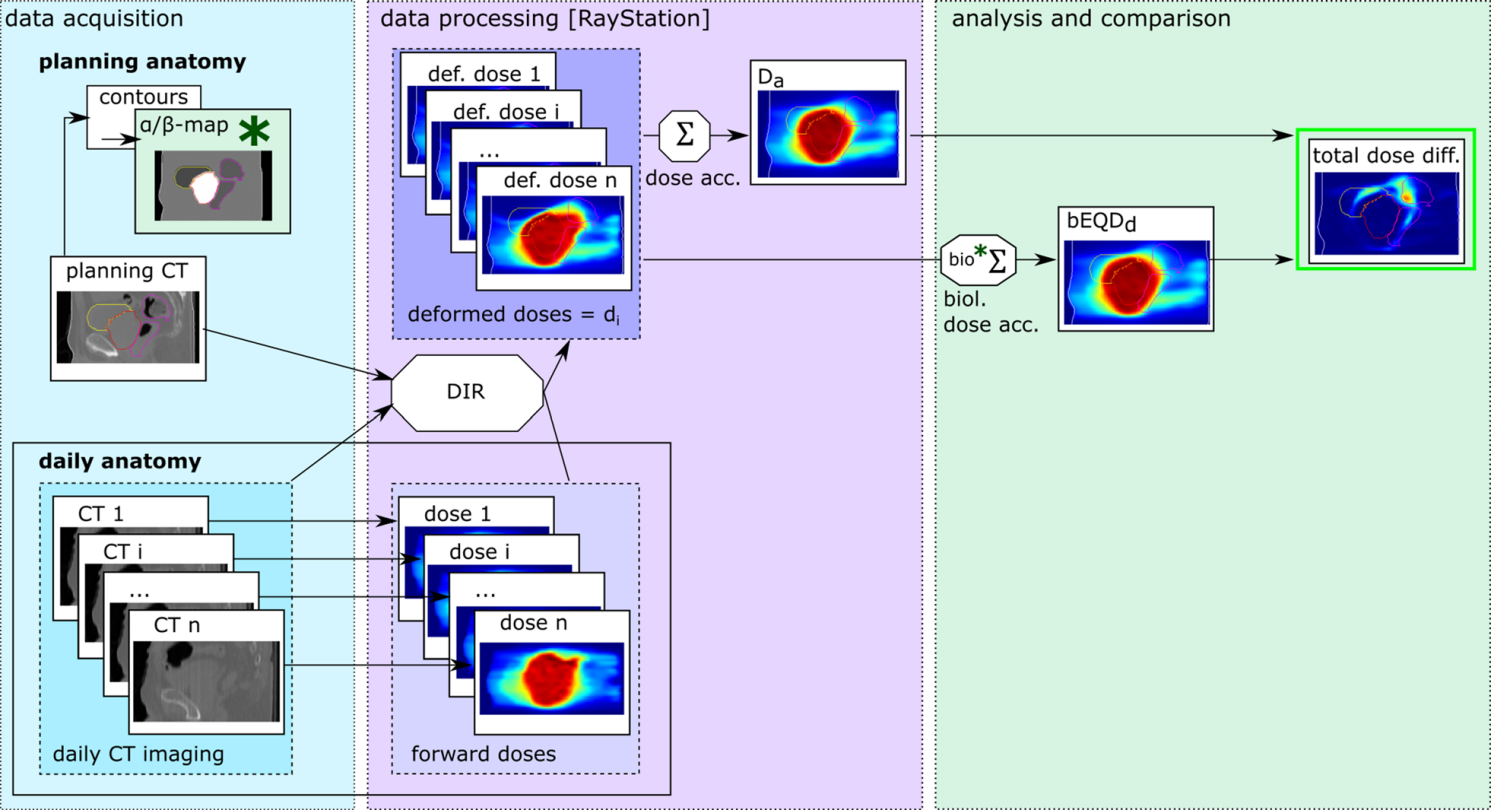
Schematic overview of the data acquisition, processing and analysis. Organ contours on the planning CT serve as basis for α/β-maps. Daily CTs are acquired in addition to the planning CT with an in-room CT scanner. On the basis of the initial dose plan and the daily CTs, daily delivered doses are forward calculated in the RayStation®. The RayStation®-integrated deformable image registration (DIR) is used to non-rigidly register the daily CTs to the planning CT. The derived deformation vector fields are then used to deform the forward calculated doses to compute the delivered dose in the anatomical frame of the planning CT (deformed delivered doses d_i_). Using the individual d_i_, the total dose is accumulated (D_a_) in the RayStation®. On the same basis of d_i_, bEQD_d_ is calculated (using the α/β-maps) according to Eq. 11. The two dose accumulation methods are compared in terms of the total dose difference (highlighted in green, top right)

Figure 1 gives a schematic overview of the data acquisition and processing. For dose accumulation it is necessary to transform the daily delivered doses into the anatomical geometry of the planning CT. For this, all fractional CTs are non-rigidly registered to the planning CT. The resulting deformation vector fields are applied to the delivered forward calculated dose (see Fig. 1), resulting in the deformed delivered doses d_i_, which are used for both dose accumulation methods.

Organ delineation, deformable image registration (DIR), dose mapping and conventional dose accumulation were performed in the RayStation®. The built-in DIR, based on the ANACONDA algorithm (a system that combines hybrid intensity and structure information) [26] was additionally guided by organ contours (“controlling ROIs”) for the bladder, rectum and target (PTV) on the planning CT and each daily CT. From the RayStation®, the deformed delivered 3D doses (d_i_) as well as the resulting conventionally accumulated dose (D_a_) were exported and used for further analysis. For additional computations of bEQD_d_, a workflow within the AVID framework (analysis of variations in interfractional radiotherapy), an in-house automation software for handling large collections of patient data, was implemented (described and used for example in [27, 28], open release in planning). Uncertainty assessments and statistical analysis were performed in R [21]. Visualization of CT and dose images was done using the software MITK [29].

To compute the SF and the biological dose, α/β-values were assigned to the contoured organs. For this, organ masks were created based on the organ contours from the planning CT using an implementation in the RTToolbox [30]. These were assigned organ-specific α/β-values to create α/β-maps. The standard α/β-map used: rectum—3.0 Gy [31], bladder—5.0 Gy [32], CTV—1.4 Gy [24], all other tissues—3.0 Gy.

### Uncertainty assessment

Both D_a_ and bEQD_d_ are prone to uncertainties inherited from the uncertainties in the image registration process. The registration outcome is dependent on the choice of DIR algorithm. Common measures of the quality of image registration address the quality of the whole deformed organ structure (e.g. Dice similarity coefficient, Hausdorff distance). Other measures only give a qualitative idea of the registration outcome that would yet need to be interpreted in terms of the registration quality (e.g. Jacobian determinant, inverse consistency). In this work, we wanted to directly compare the dependence of the total dose uncertainty from the DIR uncertainties on the respective accumulation strategies independently of the choice of DIR algorithm, and on a voxel scale. Therefore, we addressed the impact of the quality of registration by a worst-case dose mapping estimation based on a hypothetical image registration error which is equal for D_a_ and bEQD_d_. For this, we assumed a hypothetical registration error of 3 mm for each voxel in each fraction based on reports in literature [33]. To translate this to a dose mapping error, the highest dose difference within this 3 mm distance to the respective voxel was used. It should be noted that this estimation does not include any biomechanical modelling of voxel motion or similar and is a simplified dose error propagation estimation. The resulting dose difference gives a worst case estimated dose error for each voxel of each fraction denoted as Δd_i_. Standard error propagation was used with the individual Δd_i_ treated as statistically independent from each other. Following this, the registration errors propagated to D_a_ and bEQD_d_ are:

13 $$\Delta {D}_{a}\left(\Delta {d}_{i}\right)= \sqrt{{\sum }_{i}{(\Delta {d}_{i})}^{2}}$$

14 $$\Delta b{EQD}_{d}\left(\Delta {d}_{i}\right)= \frac{\sqrt{\sum_{i}{\left(\frac{\alpha }{\beta } \Delta {d}_{i} + 2 {d}_{i}\Delta {d}_{i}\right)}^{2}}}{\alpha /\beta +d}$$

In contrast to D_a_, bEQD_d_ implies another source of uncertainty which is given by the assigned α/β-values, for which a range of estimates can be found in literature. For the assessment of the uncertainty of bEQD_d_ based on the α/β-value, we calculated bEQD_d_ with different underlying α/β-maps. The variation of the α/β-values was taken from different literature sources. For the rectum, a standard value of 3 Gy was used as recommended in the QUANTEC reports [31] and values of 2 Gy and 4 Gy were used for comparison. For the bladder, a range of 5–10 Gy is given by Joiner and van der Kogel [32]. As a standard, the lower value of 5 Gy was used and compared to 10 Gy. For prostate tumors, studies suggested a very low α/β-value [24, 34, 35] in contrast to the common approach using 10 Gy for fast proliferating tumor tissues. Miralbell et al. [24] reported a value of 1.4 Gy that we decided to use as a standard. We compared this to a bEQD_d_ calculated with α/β = 10 Gy.

## Results

### What theory predicts

**Fig. 2:**
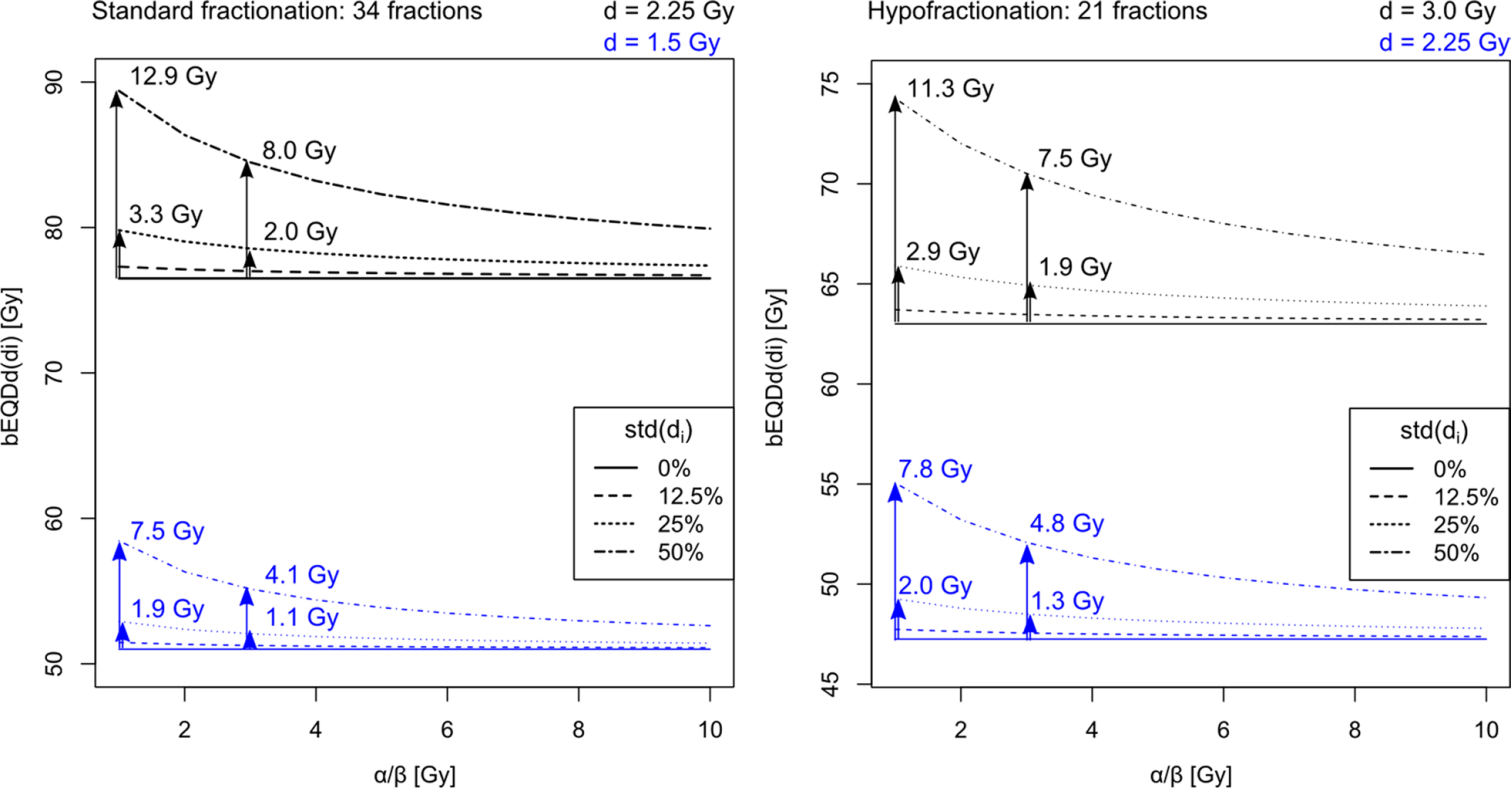
Biologically accumulated dose bEQD_d_(d_i_) for a fictional voxel receiving standard (left) or hypofractionated (right) irradiation with different magnitudes of dose variation. The dose variation magnitude is given as the standard deviation of the fractional doses d_i_ in percent with respect to the mean dose d. Note that bEQD_d_(d_i_) with std(d_i_) = 0% equals D_a_. Black curves represent the voxel in the respective high dose regions, referring to the prescription dose of 2.25 Gy (34 fractions) and 3.0 Gy (21 fractions). Blue lines represent voxels in the respective fractionated treatment in a medium dose regime, likely to occur in high dose gradients in OARs, of 1.5 Gy or 2.25 Gy. Numbers in the plot are given to show dose difference relative to the 0% dose variation case (D_a_). For less than 12.5% dose variation, the difference remains below 1 Gy

Figure 2 shows theoretical results for a fictional voxel receiving conventional and hypofractionated irradiation dose with different daily dose variation magnitudes. Three dependencies of bEQD_d_ can be seen: bEQD_d_ changing with the dose variation magnitude, given as the dose standard deviation (std), the underlying α/β-value and the fractionation scheme (number of fractions and the mean delivered dose d).

The difference between bEQD_d_ and D_a_ strongly depends on the dose variation magnitude. At a standard deviation below 12.5% of the mean physical dose, the difference between bEQD_d_ and D_a_ remains below 1 Gy. For high local dose variation magnitudes of 50% with respect to the mean physical dose, the total dose difference between the two accumulation methods can reach values of up to 12.9 Gy.

It can also be seen that the lower the α/β-value used in the bEQD_d_ calculation, the higher the difference between bEQD_d_ and D_a_. For example the highest impact in the given example can be seen for a standard fractionated treatment in the high dose regime with the highest dose variation magnitude where the difference of bEQD_d_(α/β = 1 Gy) to bEQD_d_(α/β = 10 Gy) is 9.5 Gy. This dependency induces an uncertainty in the calculation of bEQD_d_ based on the uncertainty of the α/β-value which is analyzed in the patient data results.

The curves in Fig. 2 show that for the same number of fractions, the higher the mean dose d, the higher the difference between bEQD_d_ and D_a_. Generally, the absolute differences are higher for the standard fractionated case. This is because the difference between D_a_ and bEQD_d_ accumulates over the course of treatment and scales with the total delivered dose. However, the difference between D_a_ and bEQD_d_ relative to the total delivered dose is higher for the hypofractionated case in the respective high and low dose regimes.

### What patient data show

The analysis of patient data is based on the nine patients as treated, with standard fractionation and the standard α/β-values stated above, if not indicated otherwise. One parameter or aspect was varied at a time to identify critical scenarios.

#### Comparison of bEQD_d_ and D_a_

**Fig. 3:**
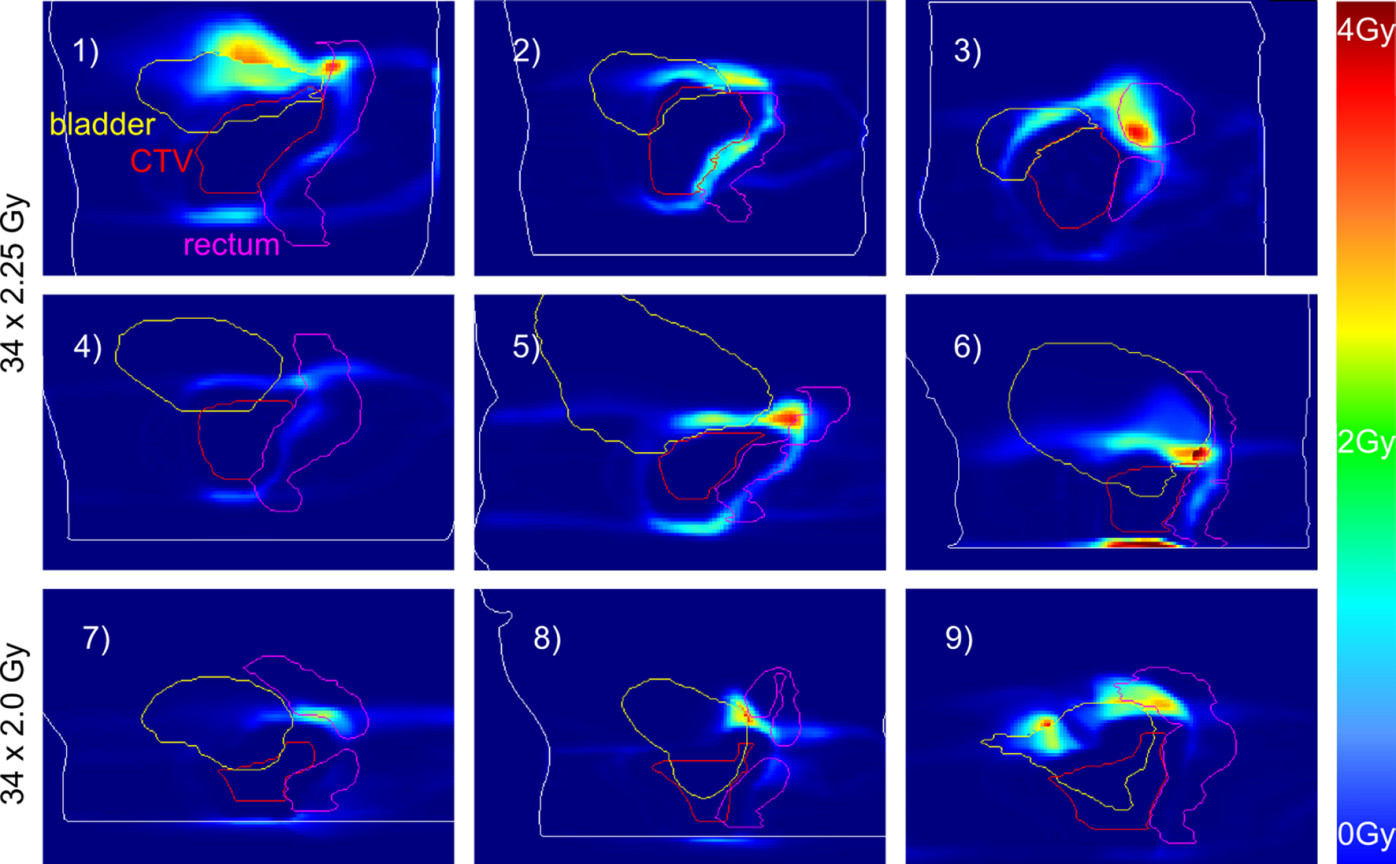
Total dose differences between the biologically accumulated dose and the conventionally accumulated dose: bEQD_d_ − D_a_ [Gy] for the nine analyzed patient cases; standard fractionation and calculated with the standard α/β-map with CTV = 1.4 Gy, rectum = 3 Gy, bladder = 5 Gy, all other tissue = 3 Gy. Contours were created by a radiation oncologist on the planning CT. As mathematically predicted, bEQD_d_ was systematically higher than (or equal to) D_a_. Each picture shows the sagittal slice with the highest dose difference for the respective patient. Cases 1)–6) represent patients treated for prostate carcinoma with the whole prostate marked as CTV; cases 7)–9) show irradiation cases of the prostatic fossa with a slightly lower prescription dose of 2.0 instead of 2.25 Gy. All cases were treated and imaged for 34 fractions. For six of the nine cases (1), 3), 5), 6), 8), 9)), differences were 3.3–4.9 Gy in distinct hot spots with a maximum difference of 4.9 Gy in case 6). Only case 4) showed differences below 1.4 Gy over the entire volume

Nine patient cases were analyzed with respect to their bEQD_d_-D_a_ difference for the standard fractionation and α/β-values (Fig. 3). In agreement with theoretical analysis, bEQD_d_ is systematically higher than (or equal to) D_a_. In all cases, larger areas with pronounced differences in total dose between bEQD_d_ and D_a_ were found in the regions of high dose gradient close to the target volume. with values around 1–3 Gy. For six of the nine patients, more distinct difference hot spots were found in the bladder and rectum, with maximum differences around 3.3–4.9 Gy in regions of high motion amplitudes. The maximum difference, of 4.9 Gy, was for case 6. For only one patient (case 4), the differences were below 1.4 Gy in the entire monitored volume. A region of repetitive high difference amplitude could be found for all patients between the delineated organs, often coinciding with the rectal and bladder wall. Discontinuities in the depicted dose differences in Fig. 3 at organ boundaries (for example in case 1 at the superior bladder boundary) are based on different underlying α/β-values for the respective tissues (e.g. α/β = 5 for the bladder and 3 for the surrounding tissue), resulting in offsets between bEQD_d_ results.

By definition, dose-difference hot spots shown in Fig. 3 coincided with hot spots of total survival overestimation by D_a_. The magnitudes of these differences depended on the total dose delivered in the respective voxel along with the dose variation magnitude. For example, the highest difference was found for case 9), where the hot spot found in the rectal wall (4.9 Gy difference between bEQD_d_ and D_a_) corresponded to a 50% difference in the survival fraction. The hot spots in cases 3), 5) and 7) corresponded to a SF difference of 35% while case 4), with the lowest difference, had a maximum of 18% SF difference.

#### Hypofractionation

**Fig. 4:**
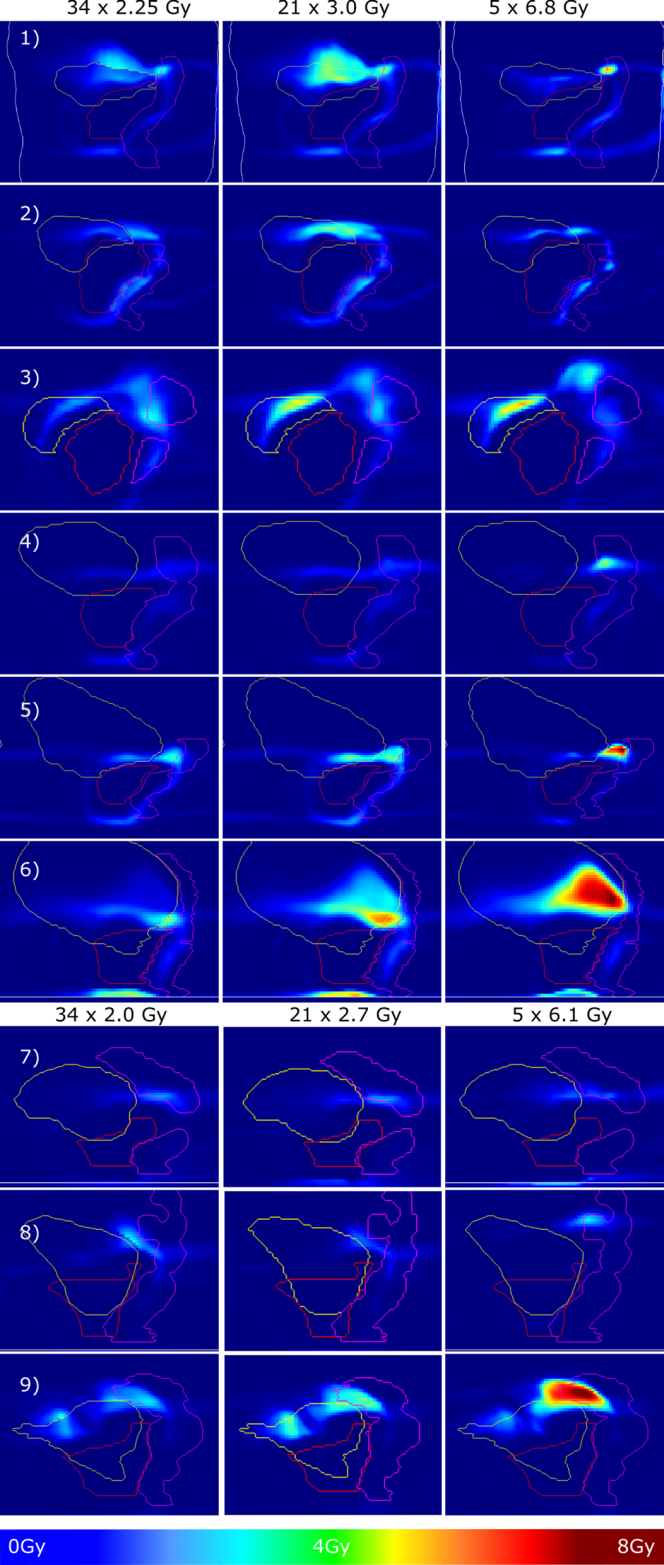
Total dose difference (bEQD_d_ − D_a_ [Gy]) for the nine analyzed patient cases (same as in Fig. 3, with standard α/β-map) using three different fractionation schemes: 34 fractions of 2.25 Gy/2.0 Gy as used for treatment, 21 fractions of 3.0 Gy/2.7 Gy (moderate hypofractionation), 5 fractions of 6.8 Gy / 6.1 Gy (strong hypofractionation). The lower 3 cases show patients treated for the prostate fossa with a slightly different dose prescription. Schemes were converted using the standard EQD formalism with prostate α/β = 1.4 Gy to be biologically equivalent. Note that the dose difference colour scaling is different to previous figures (0–8 Gy). Differences were generally increasing when going to higher fraction doses, as expected from Fig. 2. Differences were highest for 7/9 cases for strong hypofractionation (above 4.3 Gy) with a maximum of 8.4 Gy (case 9). At 21 fractions, case 6) showed the highest difference hot spot with 6.8 Gy

Figure 4 gives an overview of all 9 patient cases for all 3 analyzed fractionation schemes for the total dose difference, using the standard α/β-values. Difference magnitudes strongly increased when increasing the dose per fraction while lowering the number of fractions. Generally, hot spots in dose difference were found only in OARs within the regions of high dose gradients. In most of the cases presented, high differences were located superior to the target, at the interface between bladder wall and rectum wall. Highest differences were found for hypofractionation in 5 fractions, where 7 of 9 cases show difference hot spots above 4.3 Gy, and where there is a maximum of 8.4 Gy (case 9). The difference was above 8 Gy in 3 cases. Highest difference hot spot for the 21-fraction scheme was 6.8 Gy for case 6) with 5 cases showing difference hot spots above 5 Gy. For comparison, at 34 fractions the maximum hot spot was at 4.9 Gy. Hot spot areas are broader for the 21-fraction scheme in 7 cases and are smaller, but in most cases of higher magnitude, when the number of fractions is reduced to 5. In one case, the magnitude of difference decreased when going from 34 to 21 fractions and for two cases when going from 21 to 5 fractions.

#### Comparison with planned dose

**Fig. 5:**
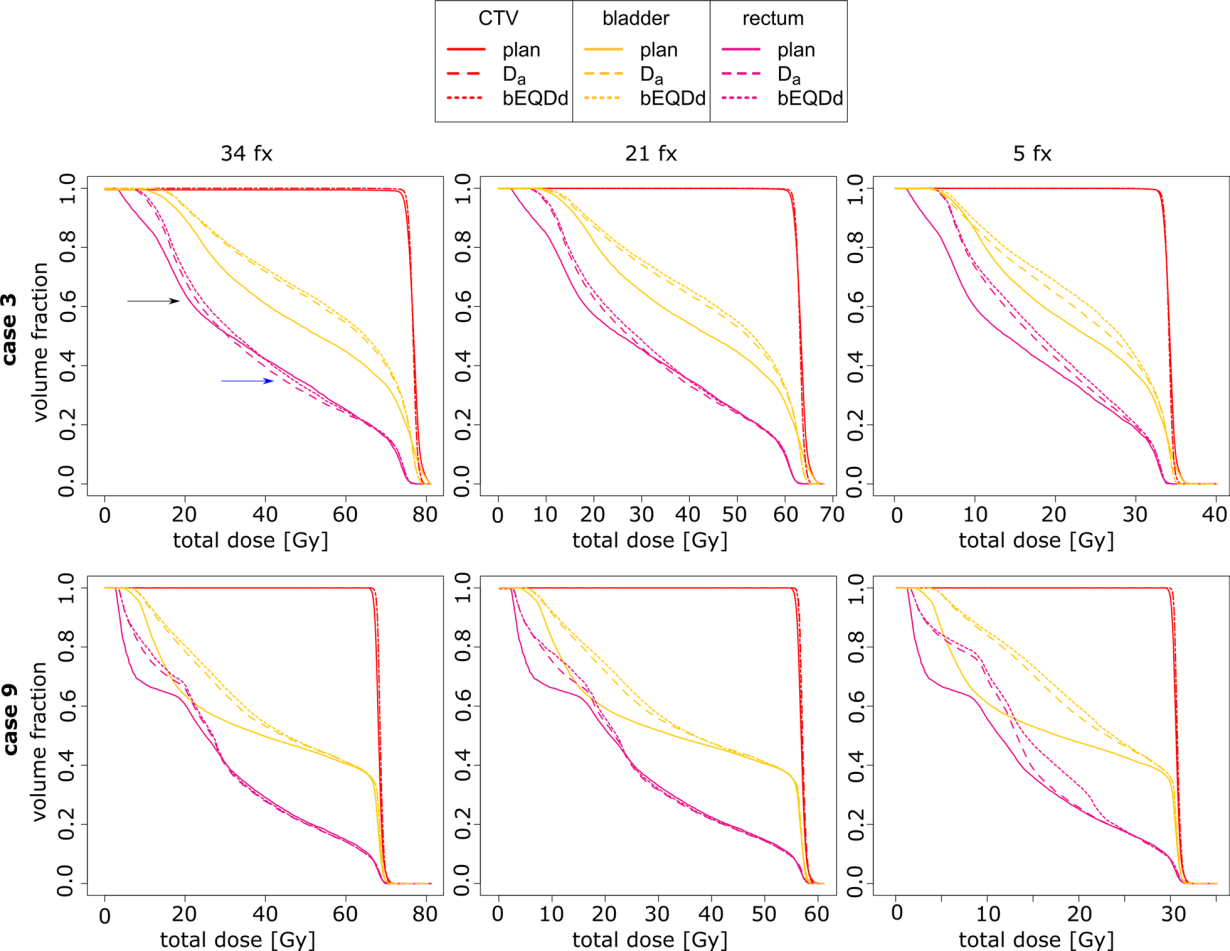
Cumulative DVHs of case 3) and 9) (see Fig. 3), showing the planned dose, the conventional accumulated dose D_a_ and the biological accumulated dose bEQD_d_ for the three investigated fractionation schemes (standard α/β-values). For case 3) the distinct hot spot difference between bEQD_d_ and D_a_ is visible only as a small difference in the curves for the rectum in the DVH in the mid dose region, while no clear difference can be seen for CTV and bladder. Examples: Black arrow—dose regime where bEQD_d_ increases the overdosage between planned and delivered dose further; blue arrow—the increase in the delivered dose calculation by bEQD_d_ is still below the planned dose. For case 9) the strong difference hot spot in the rectum is also visible as a stronger difference in the DVH for hypofractionation with 5 fractions (fx)

Dose comparison using dose volume histograms (DVHs) revealed only small differences between the two accumulation methods for standard fractionation, although distinctive hot spots of increased total dose difference are visible for all patient cases in the 3D data (Fig. 3). The DVHs of cases 3) and 9) are shown in Fig. 5. For case 3), in contrast with Fig. 3, the distinct hot spot within the volume of the rectum is visible only as a slight difference in the DVH of the rectum. There is no difference of D_a_ and bEQD_d_ for the bladder or the CTV, although for the bladder, there was a higher difference to the planned dose. The visible difference between the DVH curves of D_a_ and bEQD_d_ for the rectum were similar to the depicted case 3) for two other cases, while 6 cases showed lower difference. For both hypofractionation schemes, differences were slightly more distinct for case 3) based on the overall higher difference reported in the previous section. For case 9), the difference hot spot in the rectum for strong hypofractionation also shows a stronger difference in the DVH. The DVHs for the bladder showed generally lower differences between the two accumulation methods than those for the rectum. For both bladder and rectum, there was a distinct difference to the planned dose, similar to the depicted case in a total of three cases while the other 6 cases showed only small differences. For all cases, the dose coverage of the tumor was similar as in the depicted DVH. More information and analysis on the difference between planned and delivered dose for standard dose accumulation can be found in a preceding study [22].

Bringing the obtained results from both dose accumulation methods into a clinical context, we contrasted these with dose constraints given in the QUANTEC reports for bladder [31] and rectum [36]. We found several cases in which the constraints were violated for standard fractionation for the conventionally accumulated dose D_a._ The same parameters deviated further (in the order of 1Vol%) from the constraints in the case of bEQD_d_. This was the case for one case for bladder V_70Gy_ (case 3, QUANTEC V_70Gy_ < 35Vol%, V_70Gy_(bEQD_d_) = 41Vol%). The constraints for the rectum were violated in one case for V_50Gy_ and V_60Gy_ (case 1, QUANTEC V_50Gy_ < 50Vol%, V_50Gy_(bEQD_d_) = 53Vol%; QUANTEC V_60Gy_ < 35Vol%, V_60Gy_(bEQD_d_) = 43Vol%), and for two cases for rectum V_65Gy_, V_70Gy_ and V_75Gy_ (cases 1 and 2, QUANTEC V_65Gy_ < 25Vol%, case 1 V_65Gy_(bEQD_d_) = 37Vol%, case 2 V_65Gy_(bEQD_d_) = 30Vol%; QUANTEC V_70Gy_ < 20Vol%, case 1 V_70Gy_(bEQD_d_) = 31Vol%, case 2 V_70Gy_(bEQD_d_) = 26Vol%); QUANTEC V_75Gy_ < 15Vol%, case 1 V_75Gy_(bEQD_d_) = 22Vol%, case 2 V_75Gy_(bEQD_d_) = 20Vol%). The deviation in the V_xGy_ parameters from D_a_ and bEQD_d_ was generally increased for hypofractionation, going from a median of 0.5% in volume difference with a range of 1%, to a median of 1% with a range of 2% for moderate and strong hypofractionation. For case 1, a difference in V_21Gy_ of 7Vol% for the rectum (for strong hypofractionation, equivalent to V_30Gy_ for standard fractionation) and 4Vol% for the bladder was observed. Please note that these volumetric deviations are not necessarily visible in the one-slice-representation in Fig. 4.

#### Uncertainty assessment

**Fig. 6:**
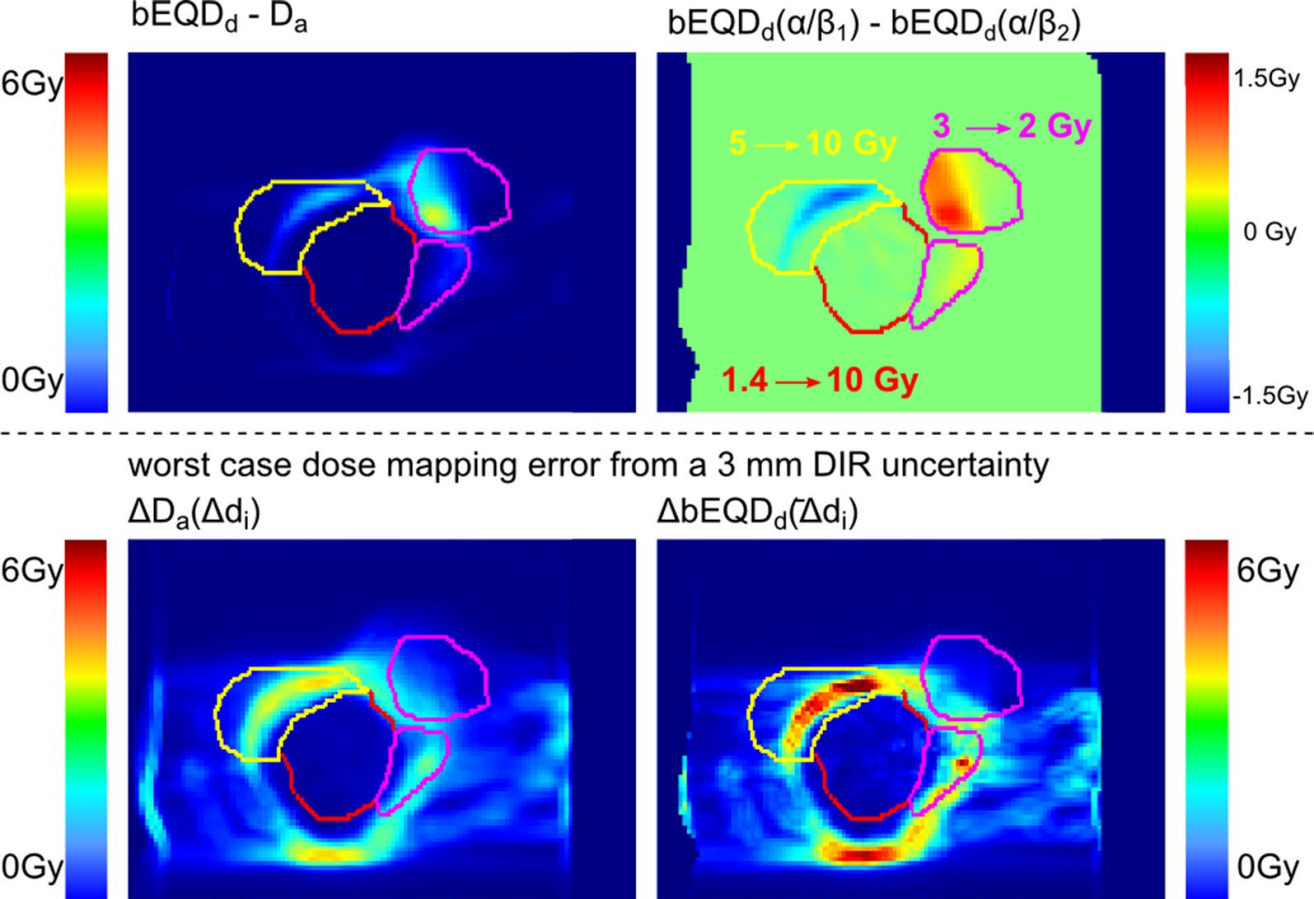
Uncertainty assessment for case 3) in standard fractionation. Top left: Difference of bEQD_d_ − D_a_ as depicted in Fig. 3; note that the scaling was changed in order to allow comparison with the bottom row. Top right: Change in bEQD_d_ induced by changing the underlying assigned α/β-values of the tissues from their standard values here to extreme values, as indicated. Note that when increasing α/β, bEQD_d_ decreases (getting closer to D_a_), while decreasing α/β (as in case of the rectum (pink) in this example), bEQD_d_ will be higher (larger difference to D_a_). As can be seen in comparison of bladder (yellow) and CTV (red), the magnitude of difference caused by a change in α/β also depended on the dose variation magnitude. Both scenarios in the upper row represent systematic differences. Bottom row: Worst case dose difference resulting from a 3 mm image registration error propagated to dose accumulation (Eqs. 13 and 14) for D_a_ (left) and bEQD_d_ (right), respectively. The bottom row is an uncertainty that is not systematic

Figure 6, bottom row, depicts the DIR worst-case error estimation for case 3) as the propagated dose error, for standard fractionation and the described standard α/β-values. Generally, it can be seen that the magnitude of uncertainty is higher for ΔbEQD_d_ than ΔD_a_. In the analysis of all patients, ΔbEQD_d_ showed maximum values around 9 Gy while the maximum of ΔD_a_ was at 5 Gy. Uncertainties are located in the gradient regions resulting from the neighboring voxel deviation estimation.

In contrast to conventional dose accumulation, biological dose accumulation depends additionally on the assigned α/β-values of the respective tissues. Generally, as reported above, the lower the α/β-value of the tissue, the higher is bEQD_d_. As an example, from patient data analysis, Fig. 6 shows the impact of changes in α/β-values for case 3) changing from the used standard values, to the most strongly differing values described in literature. Highest difference between bEQD_d_ computed with different α/β-maps coincided with regions of highest differences to D_a_. Changing the α/β-value for the rectum from 3 to 4 Gy resulted in a maximum change of 0.6 Gy (0.2 – 1.3 Gy) in bEQD_d_ averaged over all patients. Lowering α/β from 3 to 2 Gy led to a maximum increase in bEQD_d_ of 1.0 Gy (0.4–1.3 Gy). For the bladder, the difference between the lowest recommended α/β-value of 5 Gy to the highest of 10 Gy resulted in a maximum difference of 0.9 Gy (0.3–1.5 Gy). In the CTV, changes were generally lower due to the overall lower dose variation magnitude. Assigning the standard α/β-value for tumors of 10 Gy instead of the more recently promoted low α/β-value of around 1.4 Gy changed bEQD_d_ by a maximum of 0.7 Gy (0.0–1.7 Gy).

## Discussion

We introduced and evaluated the concept of the biological total dose bEQD_d_ in comparison to conventional dose accumulation and effect prediction. We showed that averaging of the daily delivered varying voxel doses by dose accumulation introduces a systematic underestimation of the total dose with respect to the resulting biological effect. This inaccuracy can be avoided with the use of bEQD_d._ In this analysis, we identified scenarios where the difference between the conventional dose accumulation approach and the biological approach presented here are most critical, and scenarios where it can be neglected. For the nine patient cases analyzed, we detected difference hotspots around 4 Gy in five cases between bEQD_d_ and D_a_ when using standard fractionation of 76.5 Gy in 34 fractions. These differences were increased to over 8 Gy when changing to a hypofractionated treatment scheme in only 5 fractions. Highest differences were localized around the target volume in the regions of high dose gradient within the OARs. The resulting dose differences depended strongly on the location and magnitude of organ motion, and was therefore highly case-specific. In most cases high differences were observed superior of the target, at the walls of bladder and rectum.

The approach presented here is similar to the equivalent total dose introduced by Zavgorodni et al. [5]. In contrast with results presented by de Xivry et al. [7], who performed an analysis using the equivalent constant dose for head and neck cancer patients, we found generally higher local differences. Their highest difference was 2.6 Gy, a value exceeded in six of our nine cases, where there was a maximum difference of 4.9 Gy in standard fractionation, and of 8.4 Gy in strong hypofractionation, in our analysis. The generally higher difference we found could be caused by an overall higher organ motion amplitude in the pelvic region compared with the head and neck cases or by the detection of such by daily imaging in contrast to weekly imaging used by de Xivry et al. [7], or the combination of both. The magnitude and differences of these findings suggest that it is of interest to perform further investigation with a higher number of cases and different tumor sites.

The need for further investigation is underlined by the theoretical predictions. These showed that the magnitude of difference between the two dose accumulation principles depends strongly on the local circumstances, and in patient data can therefore exceed the highest dose difference observed here by a factor of two. For very low dose variation magnitudes, the difference between bEQD_d_ and D_a_ is expected to be marginal (below 1 Gy for dose variation below 12.5%), which is in accordance with the theoretical predictions of Bortfeld et al. [6], who used the equivalent uniform dose principle. High dose variation magnitudes as analyzed theoretically (Fig. 2) can only be expected to occur in high dose gradients in combination with organ motion in the same region. In this case, differences can exceed 10 Gy locally. Nevertheless, in regions of homogeneous dose and/or low organ motion, where little dose variation is observed, the difference between bEQD_d_ and D_a_ remains negligible.

In the current study, we observed local daily dose variations with magnitudes between 0 and 0.5 Gy (daily dose) in the majority of voxels in bladder and rectum. Due to the voxels being located in the regions of high dose gradient, these variations correspond to percentage differences to the planned dose around 20% in the mid-dose regime (e.g. 1.4 ± 0.3 Gy in the bladder wall), increasing to 100% in low dose voxels. It should, however, be noted that the high percentage differences in the low dose regime have a lower impact on the total absolute dose, also for the bEQD_d_ analysis, as also shown by the theoretical results.

For the use of the effect accumulation approach and bEQD_d_, the same basic information is necessary as for conventional dose accumulation. For the biological effect, only the steps of accumulation and model application have to be interchanged. With the only addition of assigning the α/β-values to the respective tissues, there is no further computational effort in the calculation of bEQD_d_ compared with the accumulated dose D_a_. The bEQD_d_ approach presented here is based on the basic form of the LQM. When dealing with very low dose regions, or when strong hypofractionation, non-standard dose-rate schemes or multiple fractions a day are applied, it might be necessary to include further models or correction terms to the LQM. For the cases presented, the analyzed dose regimes are in the range for which use of the LQM in its basic form is recommended [37].

We found that the difference in total dose induced by biological dose accumulation for late responding tissues, which have low α/β-value, impacts results more than for those with a high α/β-value. This was also reported by Zavgorodni et al. [5] for the equivalent uniform dose. This is due to the impact of fractional dose variation on β rather than on α. Therefore, late responding organs like the spinal cord, colon and the cases of bladder and rectum presented here are most interesting for the application of bEQD_d_.

The difference between the two accumulation methods seems to scale with the total delivered dose and is therefore higher for standard fractionation with a high total dose but also for voxels in higher dose regions. From an algebraic point of view, this is based on a higher dose variance. This leads to an increased risk of total effect underestimation in normal tissue for hypofractionated cases as well, based on the higher prescription dose and potentially steeper dose gradients. Furthermore, higher doses further increase the difference in the dose accumulation methods since the difference is given in the β-term that is quadratic to dose. We found that this effect is dominating, leading to higher difference amplitudes for hypofractionated treatment. In rescaling the dose to moderate hypofractionation with 3.0 Gy (21 fractions) and strong hypofractionation with 6.8 Gy (5 fractions) we found that the difference magnitude increased strongly in the majority of cases, again with highest difference in dose gradient regions in OARs. Going to moderate hypofractionation, highest differences increased to 6.8 Gy (compared with 4.9 Gy in 34 fractions). For strong hypofractionation, three cases already showed a total dose difference above 8 Gy which is already 24% of the tumor prescription dose. This is in agreement with the theoretical predictions. One should note that the comparison of the total dose difference for different fractionation schemes is not only influenced by the choice of the treatment dose, but also by the “choice” of the fractions that are taken into account in the presented analysis. It might be that by lowering the number of fractions, this might exclude fractions of high motion amplitude present in the analysis with more fractions. Conversely, high motion amplitudes occurring in the included fractions have a greater weight. Thus, differences might decrease when lowering the number of fractions in case high motion amplitudes occurred more often in fractions that were not taken into account which was observed for 3 cases. It should also be noted that scaling of the dose was chosen over setting up new hypofractionated treatment plans to ensure a more direct comparison of the total dose difference. Scaling was performed on already deformed doses. The observed difference is then based only on the accumulation method and the above-mentioned fractions that are included. We found that with new plans, resulting differences can change a lot in hotspot magnitude and location based on different beam weights that might or might not traverse areas of higher or lower motion amplitude.

While the difference between the two dose accumulation strategies is based on the magnitude of dose variation from day to day, this does not necessarily coincide with larger systematic deviations from the planned dose. For example, the patient’s bladder might have been larger during the planning CT than in any other fraction, leading to a systematic difference from the planned dose, but with small day-to-day dose variations. This provides a possible explanation for the DVH of case 3, presented in Fig. 5, where there is little difference between bEQD_d_ and D_a_ for the bladder but a higher difference from the planned dose. For the rectum, there may have been larger day-to-day motion, leading to the difference hot spot and DVH difference shown, but the total dose was lower than the planned dose.

The small differences between the DVHs of bEQD_d_ and D_a_ (Fig. 5), for standard fractionation, indicate that when clinical decisions or dose-response modelling are solely linked to a few distinct DVH-parameters, it is likely that there will be no change in outcome when using one or the other dose accumulation strategy in standard fractionated treatment. The impact will be increased in hypofractionated cases, with generally higher dose differences also visible in DV-parameters. This is in agreement with conclusions by de Xivry et al. [7]. In comparison to Fig. 3 and Fig. 5, however, this highlights the limitation of the use of dose-to-volume-based simplifications of the 3D dose information and is a serious over-simplification of the spatial dose distribution for finding correlations between dose and toxicity. Taking into account that dose differences tend to be local rather than global, the impact of using bEQD_d_ rather than D_a_ has greater importance for serial-type organs, such as the spinal cord or rectum. Results were compared with the given constraints for bladder and rectum doses for standard fractionation from the QUANTEC reports [31, 36]. bEQD_d_ only increased the V_xGy_ parameters by a small amount, and cases that violated the constraints were already observed for D_a_ alone. However, the differences between the dose-volume parameters from bEQD_d_ and D_a_ were increased for hypofractionation for which no dose constraints are given from the QUANTEC reports. Individual cases of V_xGy_ deviations of the order of several percent indicate that clinical decisions might be changed by the use of bEQD_d_. This was especially the case for dose to the rectum in the mid-dose regimes.

Currently, more studies focus on the use of local dose information rather than dose volume histograms [8–11]. Shelley et al. [4], for example, found higher correlation with toxicity when using daily dose information and dose surface maps of the rectum. Using the bEQD_d_ approach in combination with local dose analysis for toxicity prediction would be a highly interesting investigation and might further increase the predictive power of the analysis.

We therefore recommend the consistent use of the biological model, facilitated by bEQD_d_, wherever daily dose information is used on a local scale, especially for doses in serial-type late-responding organs at risk. An important application might be dose-response modeling, especially for cases where dose surface maps or organ sub-regions are investigated. Furthermore, the biological dose accumulation might result in different conclusions in the process of treatment replanning and adaptation when, for example, dose constraints might be violated at an earlier stage due to the systematically higher total dose. Another application is in retreatment of patients. Boman et al. [38] investigated cases of retreatment using deformable image registration and the use of the EQD_2_ in order to estimate the (biological) dose already applied, and found difference of more than 10 Gy locally between undeformed and deformed (biological) dose. Daily imaging and application of bEQD_d_ might further change this number and might therefore change decisions for the retreatment plans.

### Uncertainty assessment

The difference between bEQD_d_ and D_a_ is as a systematic error in the post-processing of the dose data with respect to the biological effect. It can therefore itself be interpreted as an inaccuracy in the overall accumulated dose. Thus, bEQD_d_ increases the accuracy of biological effect prediction. Nevertheless, both bEQD_d_ and D_a_ are subject to uncertainties of their own. Above all, the uncertainties in deformable image registration (DIR) propagate to the mapped voxel doses. Propagation of DIR uncertainties to the accumulated dose has been a popular field of research due to its increasing importance for emerging methods [39–42] and there is not yet a standardized quality assurance procedure available. It is beyond the scope of this work to perform an in-depth analysis of the DIR accuracy. This uncertainty will strongly depend on the choice of the underlying DIR algorithm, parametrizations and constraints. We chose to use commercially approved DIR software implemented in the RayStation®. Furthermore, organ contours were used to guide the DIR to improve registration quality. Nevertheless, image registration errors, especially within an organ of homogeneous imaging values, can occur. Image registration is arguably the biggest source of uncertainty in the dose accumulation procedure and has been a controversial topic [43]. This, however, holds true for both D_a_ and bEQD_d_.

Here, we focused solely on the difference in the impact of dose mapping errors on the accumulation strategies, independently of the choice of the DIR algorithm. To allow a more general estimate of the impact of possible registration errors, a worst-case dose mapping error was estimated, and was then propagated to D_a_ and bEQD_d_. We chose a worst-case DIR displacement error of 3 mm, in accordance with findings in literature [33]. This is, however, a worst-case estimation and should be regarded as such. Organs might show bulk movement as well as complex motion interplay between adjacent voxels, not considered in this simplified estimation. Overall, and especially at organ boundaries, often located in the regions of high dose gradient around the target, we expect lower errors in the mapped doses than estimated here.

Of higher interest is the difference between the propagated errors in bEQD_d_ and D_a_. Although the underlying dose error was equal, its propagation depends on the accumulation strategy. As a result, the estimated uncertainties for bEQD_d_ were up twice those of D_a_. Thus, results suggest that registration uncertainties are greater in bEQD_d_ than in D_a_. This could mean in summary that the precision of D_a_ is higher than that of bEQD_d_, while bEQD_d_ has a higher accuracy (due to the systematic error in D_a_). However, we argue here that the underestimation of the total dose given by D_a_ compared with bEQD_d_ discussed above, holds true as well for the uncertainty estimation in dose accumulation. The averaging of the daily doses in D_a_ as well as the daily dose errors (Eq. 13) leads to an underestimation of the total dose as well as of the total dose error. Therefore, not only is the accuracy of D_a_ limited due to a systematic underestimation, but also its precision is expected to be lower than what simple error propagation suggests. The systematic differences of D_a_ can be prevented by using bEQD_d_, nevertheless, in order to increase the precision of both, the underlying DIR needs to be improved and its quality quantified.

The assignment of the α/β-value to compute bEQD_d_ represents a limitation to the accuracy of the total biological dose that is not present for D_a._ It is however manifested in the same way for the SF of the dose and the effect accumulation approach. The induced uncertainty in bEQD_d_ is smaller than the potential registration uncertainty or the difference to D_a_. Due to its potential magnitude of more than 1 Gy, however, it needs to be considered in the overall uncertainty budget. The magnitude of bEQD_d_ uncertainty depends on the uncertainty in α/β and is therefore organ specific, and can be improved by a higher precision in the α/β-value itself.

In summary, the magnitude of the estimated uncertainties is in the same range as the difference between bEQD_d_ and D_a_. This highlights two issues: first, the use of dose accumulation is generally prone to high uncertainties from several sources that need to be quantified and taken into account for both D_a_ and bEQD_d_; second, the biological inaccuracy in dose accumulation has as strong an impact on the overall uncertainty as other highly investigated sources such as DIR. In contrast to the DIR uncertainty, it can however be easily avoided by use of bEQD_d_.

## Conclusion

The total biological dose bEQD_d_ can be used to overcome a systematic inaccuracy in the prediction of the biological effect in radiotherapy treatments induced by conventional dose accumulation. The difference between the two accumulation strategies manifests locally on a scale of several Gy that can be clinically relevant in organs at risk and is highly increased for hypofractionated treatment. On a dose-volume level differences are marginal for standard fractionation but can impact clinical decisions in hypofractionation. Differences between the two methods are negligible only in case where daily dose variations are small (< 12.5% int the analysis presented here), which underlines the importance of daily dose monitoring. The application of bEQD_d_ is of higher interest in the context of dose-response modelling on a local scale, for example when using dose-surface maps. Highest impact is to be expected for serial-type late-responding organs at risk, undergoing frequent organ motion in regions of high dose gradient. To further improve both accuracy and precision of the total biological dose, better estimates of the tissue α/β-values are necessary and DIR uncertainties need to be quantified and reduced. Our results suggest that the total biological dose should be investigated and used also for other tumor sites and larger patient cohorts for the prediction of toxicity, in treatment adaptation and retreatment. The occurrence of individual cases showing high differences between the standard dose accumulation approach and bEQD_d_ indicate that this approach might be especially important in treatment adaptation.

## Data Availability

None.

## Abbreviations

bEQD_d_: Biological equivalent dose, total biological dose
CT: Computed tomography
CTV: Clinical target volume
d: Dose per fraction
D_a_: Total (accumulated) dose
d_i_: Daily dose
D_p_: Total planned dose
DA: Dose accumulation
DIR: Deformable image registration
DVH: Dose volume histogram
E: Biological effect
fx: Fractions
Gy: Gray [dose unit]
IMRT: Intensity modulated radiation therapy
LQM: Linear quadratic model
n: Number of fractions
SF (cell): Survival fraction
V_50Gy_: Volume receiving at least 50 Gy (equivalent for any other given dose)
